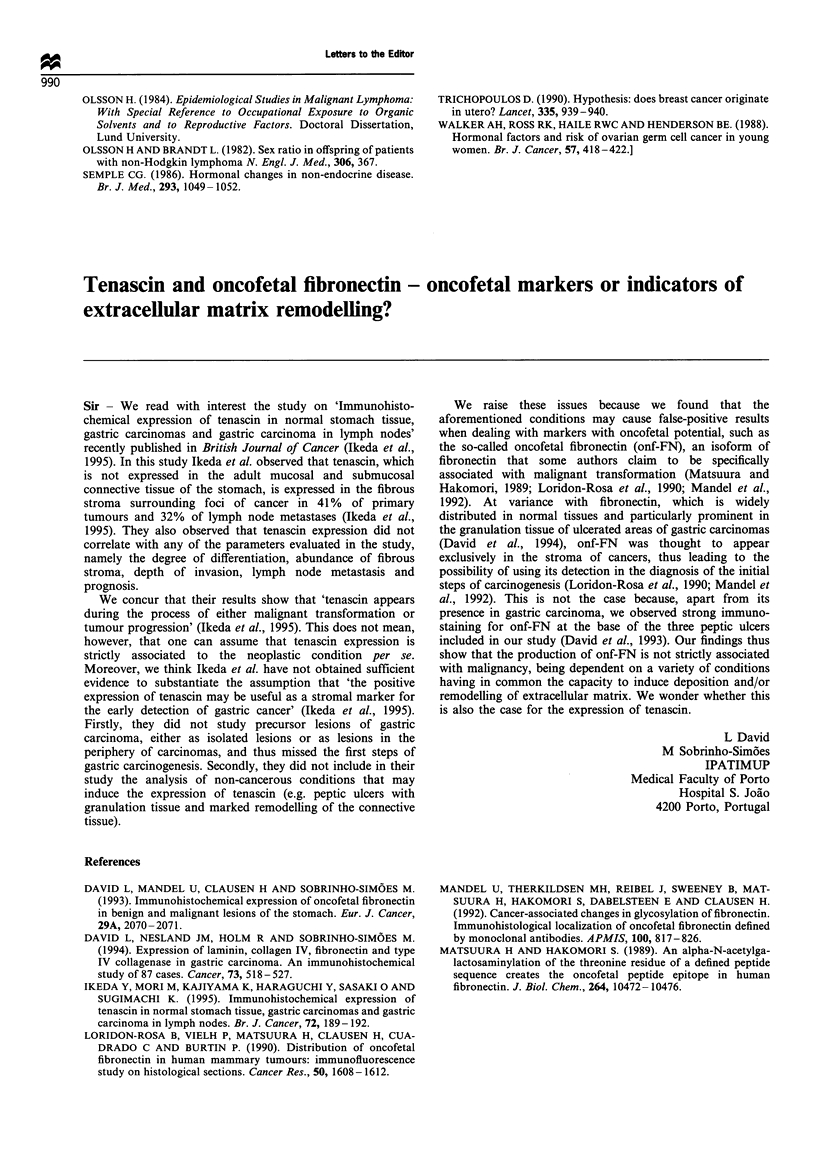# Tenascin and oncofetal fibronectin--oncofetal markers or indicators of extracellular matrix remodelling?

**DOI:** 10.1038/bjc.1996.472

**Published:** 1996-09

**Authors:** L. David


### Tenascin and oncofetal fibronectin – oncofetal markers or indicators of extracellular matrix remodelling?

**Sir** – We read with interest the study on 'Immunohistochemical expression of tenascin in normal stomach tissue, gastric carcinomas and gastric carcinoma in lymph nodes' recently published in *British Journal of Cancer* (Ikeda *et al.*, 1995). In this study Ikeda *et al.* observed that tenascin, which is not expressed in the adult mucosal and submucosal connective tissue of the stomach, is expressed in the fibrous stroma surrounding foci of cancer in 41% of primary tumours and 32% of lymph node metastases (Ikeda *et al.*, 1995). They also observed that tenascin expression did not correlate with any of the parameters evaluated in the study, namely the degree of differentiation, abundance of fibrous stroma, depth of invasion, lymph node metastasis and prognosis.

We concur that their results show that 'tenascin appears during the process of either malignant transformation or tumour progression' (Ikeda *et al.*, 1995). This does not mean, however, that one can assume that tenascin expression is strictly associated to the neoplastic condition *per se*. Moreover, we think Ikeda *et al.* have not obtained sufficient evidence to substantiate the assumption that 'the positive expression of tenascin may be useful as a stromal marker for the early detection of gastric cancer' (Ikeda *et al.*, 1995). Firstly, they did not study precursor lesions of gastric carcinoma, either as isolated lesions or as lesions in the periphery of carcinomas, and thus missed the first steps of gastric carcinogenesis. Secondly, they did not include in their study the analysis of non-cancerous conditions that may induce the expression of tenascin (e.g. peptic ulcers with granulation tissue and marked remodelling of the connective tissue).

We raise these issues because we found that the aforementioned conditions may cause false-positive results when dealing with markers with oncofetal potential, such as the so-called oncofetal fibronectin (onf-FN), an isoform of fibronectin that some authors claim to be specifically associated with malignant transformation (Matsuura and Hakomori, 1989; Loridon-Rosa *et al.*, 1990; Mandel *et al.*, 1992). At variance with fibronectin, which is widely distributed in normal tissues and particularly prominent in the granulation tissue of ulcerated areas of gastric carcinomas (David *et al.*, 1994), onf-FN was thought to appear exclusively in the stroma of cancers, thus leading to the possibility of using its detection in the diagnosis of the initial steps of carcinogenesis (Loridon-Rosa *et al.*, 1990; Mandel *et al.*, 1992). This is not the case because, apart from its presence in gastric carcinoma, we observed strong immunostaining for onf-FN at the base of the three peptic ulcers included in our study (David *et al.*, 1993). Our findings thus show that the production of onf-FN is not strictly associated with malignancy, being dependent on a variety of conditions having in common the capacity to induce deposition and/or remodelling of extracellular matrix. We wonder whether this is also the case for the expression of tenascin.

L David
M Sobrinho-Simões
IPATIMUP
Medical Faculty of Porto
Hospital S. João
4200 Porto, Portugal

### References

DAVID L, MANDEL U, CLAUSEN H AND SOBRINHO-SIMÕES M. (1993). Immunohistochemical expression of oncofetal fibronectin in benign and malignant lesions of the stomach. *Eur. J. Cancer*, **29A**, 2070–2071.
DAVID L, NESLAND JM, HOLM R AND SOBRINHO-SIMÕES M. (1994). Expression of laminin, collagen IV, fibronectin and type IV collagenase in gastric carcinoma. An immunohistochemical study of 87 cases. *Cancer*, **73**, 518–527.
IKEDA Y, MORI M, KAJIYAMA K, HARAGUCHI Y, SASAKI O AND SUGIMACHI K. (1995). Immunohistochemical expression of tenascin in normal stomach tissue, gastric carcinomas and gastric carcinoma in lymph nodes. *Br. J. Cancer*, **72**, 189–192.
LORIDON-ROSA B, VIELH P, MATSUURA H, CLAUSEN H, CUADRADO C AND BURTIN P. (1990). Distribution of oncofetal fibronectin in human mammary tumours: immunofluorescence study on histological sections. *Cancer Res.*, **50**, 1608–1612.
MANDEL U, THERKILDSEN MH, REIBEL J, SWEENEY B, MATSUURA H, HAKOMORI S, DABELSTEEN E AND CLAUSEN H. (1992). Cancer-associated changes in glycosylation of fibronectin. Immunohistological localization of oncofetal fibronectin defined by monoclonal antibodies. *APMIS*, **100**, 817–826.
MATSUURA H AND HAKOMORI S. (1989). An alpha-N-acetylgalactosaminylation of the threonine residue of a defined peptide sequence creates the oncofetal peptide epitope in human fibronectin. *J. Biol. Chem.*, **264**, 10472–10476.